# An Epileptic Colony for Texas

**Published:** 1899-06

**Authors:** 


An Epileptic Colony for Texas.—One of the few sensible acts of the Twenty-sixth Texas Legislature, which has just adjourned, was the passage of a bill creating an asylum for epileptics in Texas. I doubt if this would have been done but for the fact that it was "a platform demand," and of course all candidates on the democratic ticket had to pledge themselves to it. Taylor county donated a large tract of land near Abilene for the purpose, and the Legislature appropriated $100,000 for erecting suitable buildings and other improvements, and $1000 to pay the expenses of an expert to visit established colonies for epileptics in the North and investigate the details of operation, the style of buildings, the management, etc., and then submit plans for the Texas asylum. The Governor appointed to this important duty Dr. B. M. Worsham, the Superintendent of the Texas Insane Asylum at Austin. This is an excellent appointment. Dr. Worsham is a skilled alienist and a thorough asylum man. He was Assistant Superintendent of the Austin Insane Asylum four years under Hogg's administration, Superintendent four years under Culberson's, and was re-appointed Superintendent by Governor Sayers. Dr. Worsham left Austin June 1st to visit the now famous "Craig Colony" in New York, and the State Epileptic Colonies in Massachusetts and Pennsylvania. On his return plans and specifications will at once be submitted to the Governor, and the work of improving the grounds will begin at once. The neglect of the epileptics in Texas had become a standing reproach to the State, and it is with much gratification that the JOURNAL announces that the evil is to be at once corrected. In this connection we will add that appropriations were made by the Legislature also for enlarging the capacity of the several insane asylums. Superintendent Wilson of the North Texas Insane Asylum at Terrell informed the writer that last year four hundred applicants for admission to the asylum were turned off for lack of room, and they were mostly women. Still, with all the additional room provided, there will be a large number of these unfortunates who must still stay in jail because of the State's lack of provision for them in asylums.

